# Using machine learning to investigate earning capacity in patients undergoing psychosomatic rehabilitation—A retrospective health data analysis

**DOI:** 10.3389/fpsyt.2022.1039914

**Published:** 2022-10-20

**Authors:** Lilia Papst, Volker Köllner

**Affiliations:** ^1^Psychosomatic Rehabilitation Research Group, Department of Psychosomatic Medicine, Center for Internal Medicine and Dermatology, Charité University Medicine Berlin, Berlin, Germany; ^2^Department of Psychosomatics and Behavioural Psychotherapy, Rehabilitation Centre Seehof, Teltow, Germany

**Keywords:** disability, psychiatric disorders, earning capacity, machine learning, early retirement, gradient boosted model

## Abstract

Psychiatric disorders increasingly contribute to disability and early retirement. This study was conducted to investigate whether machine learning can contribute to a better understanding and assessment of such a reduced earning capacity. It analyzed whether impaired earning capacity is reflected in missing treatment effects, and which interventions drive treatment effects during psychosomatic rehabilitation. Analyses were based on routine clinical data encompassing demographics, diagnoses, psychological questionnaires before, and after treatment, interventions, and an interdisciplinary assessment of earning capacity for *N* = 1,054 patients undergoing psychosomatic rehabilitation in 2019. Classification of patients by changes in self-reported mental health and interventions predictive of changes were analyzed by gradient boosted model. Clustering results revealed three major groups, one of which was comprised almost exclusively of patients with full earning capacity, one of patients with reduced or lost earning capacity and a third group with mixed assessments. Classification results (Kappa = 0.22) indicated that patients experienced modestly divergent changes over the course of rehabilitation. Relative variable influence in the best model was highest for changes in psychological wellbeing (HEALTH-49). Regression analysis identified intervention A620 (physical exercise therapy with psychological goal setting) as most influential variable predicting changes in psychological wellbeing with a model fit of *R*^2^ = 0.05 (*SD* = 0.007). Results suggest that disability due to psychiatric disorders does associate with distinct demographic and clinical characteristics but may be less clear-cut in a subgroup of patients. Trajectories of treatment response show moderately divergent paths between patient groups. Moreover, results support both physical exercise therapy as efficient intervention in reducing disability-associated impairments and the complementarity of a multimodal treatment plan.

## Introduction

Psychiatric disorders are among the lead causes for disability and premature retirement both in Europe and worldwide (1–3). Indeed, their prevalence has been increasing since the 90s, consistently accounting for more than 14% of age standardized years lived with disability (YLDs). Also, YLD counts were strikingly concentrated in the working-age population (4), meaning that disability due to psychiatric disorders makes for an increasingly large component of income loss and health expenditure. In 2018, 7.6% of the total expenditure for social protection benefits by EU Member States was spent on disability benefits (5). Meanwhile, disability can be considered a broad and context dependent construct, which makes its valid, reliable, and standardized evaluation challenging. The support of clinical decision-making by machine learning applications is on the rise across all medical disciplines (6) and may be a useful addition in the assessment of earning capacity.

In Germany, a full or partial disability pension can be applied for if an individual has a diagnosis, is unable to participate in the regular job market for more than 6 h a day, cannot achieve improvements through rehabilitation and is not expected to improve within a timeframe of 6 months. Patients with mental disorders are therefore referred to psychosomatic rehabilitation prior to application for disability pension (7). Psychosomatic rehabilitation treatment seeks to preserve and restore earning capacity in patients suffering from mental and physical illness and work-related problems. Indeed, these factors may intersect at multiple levels. The psychological stress associated with low socio-economic status and difficult working conditions (8) may not only cause hypothalamic-pituitary-adrenal axis activation (9, 10) and affect the (auto)immuno-inflammatory response (11–13), but moreover favor risk behaviors such as smoking, poor diet and physical inactivity. In sum, these are common risk factors in the pathogenesis of obesity, depression, cardiometabolic disorders, and diabetes (14). These illnesses may in turn compromise work ability and close the vicious cycle by adding to financial and psychosocial stress. Rehabilitants may therefore present with an array of diagnoses caused by mutually dependent psychobiological processes. The interconnectedness of these factors is addressed in psychosomatic rehabilitation by pursuing an interdisciplinary approach encompassing individual and group psychotherapy, medical examinations, pharmacotherapy, physical training, nutrition counseling, ergotherapy, creative therapies, and social counseling. Having received treatment for a minimum of 5 weeks, an evaluation of earning capacity is then made regarding both the last job held by the patient and the general labor market (7, 15). Following in-patient treatment, a rehabilitant may thus be discharged as currently fit for work or unfit for work, accompanied by an assessment of prospective earning capacity. The latter may eventually translate into a partial or full disability pension.

The aim of this hypothesis-free, data-driven approach was to assess whether different degrees of disability can be represented by patient characteristics, i.e., whether these patients belong to distinct groups with distinct features. Because a key question in the provision of disability pensions is whether successful treatment is considered possible, the analysis focused on changes in psychological variables between admission and discharge. In addition, we investigated which interventions had the greatest influence on symptom improvement. We found that disability due to mental disorders can manifest in a distinctive clinical picture but is less clear-cut in a subgroup of patients. Trajectories of change followed differing trends depending on earning capacity, although with high overlap. We further provide support for additional investigations into exercise therapy as prevention measure for mental health-associated disability.

## Materials and methods

### Patients

Analyses were based on routine clinical data of *N* = 1,054 patients undergoing psychosomatic rehabilitation who were discharged between January 1st and December 31st 2019. The regular duration of rehabilitative treatment is 5 weeks. Patients were asked for written consent on the use of clinical data for research purposes and informed about their rights to refuse data processing without indication of reasons or disadvantages to their treatment. The State Chamber of Physicians of Brandenburg was contacted for ethical consultation. An ethics statement was not required under Brandenburg state law (§10 Datenschutz bei Forschungsvorhaben)^1^ due to the retrospective analysis of health data routinely acquired within the own department, restriction of data access to third parties, as well as use of exclusively anonymized data for analyses and publication.

### Data acquisition

Data included demographics, diagnoses according to the International Classification of Diseases (ICD-10), computer-based questionnaire diagnostics before and after treatment and an interdisciplinary assessment of earning capacity (full = ≥ 6 work hours a day, partial = 3–6 work hours a day, lost = ≤ 3 work hours a day). A record of received interventions was available for *n* = 717 rehabilitants. Demographic data were part of patient admission records and included date of birth, sex, marital status, employment, and education. Additional work-related data encompassed whether patients were on sick leave, the overall duration of sick leave within the last 12 months (≤3 months, 3–6 months, ≥ 6 months) and fitness to work at discharge. Evaluation of earning capacity was based on interdisciplinary assessments in weekly team meetings discussing patient functionality within different therapy settings. Diagnoses included all diagnoses coded in the ICD-10 and were either part of the admission papers for referral to psychosomatic rehabilitation or diagnosed by medical or psychological staff during rehabilitation. Computer-assisted questionnaire diagnostics were conducted in groups of five at admission and discharge and included the Beck Depression Inventory (BDI-II) (16), Measures of Work-related Behavioral and Experiential Patterns (AVEM) (17), Hamburg Modules for the Assessment of Psychosocial Health (Health-49) (18), Abridged Cognitive Effort Scale (ACES) (19), Insomnia Severity Index (ISI-G) (20), SINUS-Milieu questionnaire for Germany (SINUS) (21), and the Adjustment Disorder New Module 20 (ADNM-20) (22). Analyses were run in R (Version 4.0.2) and R Studio (Version 1.3.959).

### Data analyses

#### Preprocessing

The data set was comprised of numeric, binary, and categorical variables. Treatment effect or change was computed from T0 and T1 measures (T1–T0). Diagnoses were coded as binary variables (0 = no, 1 = yes), factors were ordered where meaningful. The complete dataset was comprised of 1071 patient-specific variables and 120 interventions as defined by the German Federal Pension Fund classification of therapeutic services. Missingness analyses were performed by visual inspection using the VIM (Visualization and Imputation of Missing Values) package and missing data imputed with the k Nearest Neighbors algorithm using the square root of N to determine k (*k* = 32.47) (23).

#### Clustering analyses

Numeric variables were centered and scaled before clustering. Unsupervised feature selection was performed to eliminate variables with zero or near zero variance. Variables with a ratio of over 19 between the most common value and the second most common value and less than 0.1% of distinct values were filtered out of the data set resulting in 129 variables. To obtain clusters unaffected by the eventual assessment, Gower’s distance (24) between patients was computed omitting variables that indicate earning capacity. The resulting distance matrix was then passed to Kruskal’s (25, 26) non-metric multidimensional scaling (NMDS) function as implemented in the vegan package (27) for dissimilarity clustering.

For heatmap visualization, the distance matrix was used to create patient dendrograms, while variable dendrograms were computed using k means clustering. The optimal number of clusters for partitioning around medoids was determined using the average silhouette method. A high average silhouette width indicates good clustering (28).

#### Classification analysis

**FIGURE 1:**
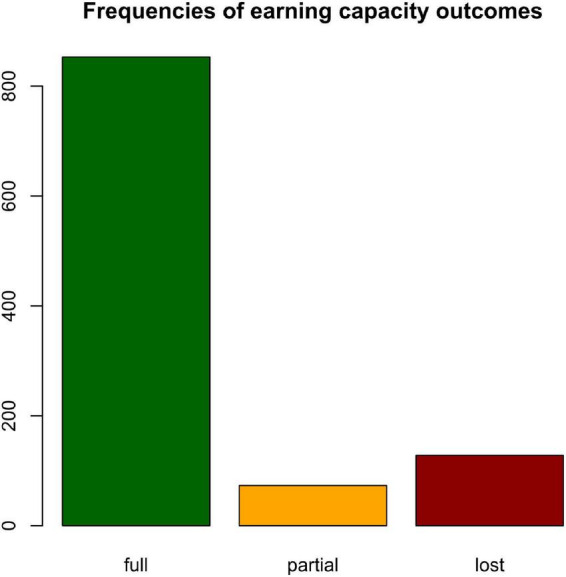
Class imbalance. Frequency distribution of earning capacity assessments in the training set.

The gradient boosted model as implemented in the caret package (29) was used to identify changes in self-reported mental health (T1–T0) predictive of earning capacity. First, variables with a mean absolute correlation of *r* ≥ 0.7 were filtered from the data set. The data was then randomly split into a training and test set by outcome (*p* = 0.6), resulting in *n* = 633 cases in the training set and *n* = 421 cases in the test set. Due to class imbalance of the outcome variable with *n* = 512 patients assessed with full earning capacity, *n* = 44 with partial earning capacity, and *n* = 77 with lost earning capacity (Figure 1), weights of 1, 8, and 6 were applied to the training set, respectively. Cross-validation (cv) was performed with *n* = 2 resampling iterations. The hyperparameter search was tuned manually using a grid with arrays of 50, 100, 500, 1,000, and 2,000 trees, interaction depths of 1, 2, 3, 4, 6, and 8, shrinkage factors 0.001, 0.005, 0.125, and 0.15, and a minimum of 2, 4, 8, 12, and 16 observations per node. No pre-processing was performed due to the robustness of tree-based models with respect to distribution. The tuning process was optimized for Kappa. Model performance was evaluated based on Kappa and the confusion matrix.

#### Regression analysis

Regression was performed to assess which interventions predicted the treatment effect differentiating strongest between evaluations of earning capacity. First, intervention data was randomly split into a training and test set (*p* = 0.5) by outcome. Predictors were evaluated with the gradient boosted model (gbm) for regression in the caret package. Cross-validation was used as training control (*n* = 2) and a tuning grid defined with possible interaction depths of 1, 2, and 3, *n* = 50, 250, 500, 1,000, and 2,000 trees, shrinkage parameters 0.001, 0.005, 0.01, 0.025 and a minimum of 2, 5, 10, and 20 observations per node. Model performance was additionally evaluated on the hold-out test set.

## Results

### Demographics

The mean age of the sample group was 51.9 years (*SD* = 8.77). Demographic and work-related data are presented in Table 1. Missingness structure is depicted in Supplementary Figure 1.

**TABLE 1:** Demographic data.

Variable	Factor level	*N*	%
Sex	Male	366	34.72
	Female	688	65.28
	Missing	0	0
Marital status	Married	553	52.47
	Single	114	10.82
	Divorced	95	9.01
	Widowed	24	2.28
	Missing	268	25.43
Education	Special needs school/none	12	1.14
	Compulsory school/no vocational training	37	3.51
	High school	272	25.81
	Vocational training	625	59.30
	University	108	10.25
	Missing	0	0
Employment	Employed	776	73.62
	Unemployed	278	26.38
	Missing	0	0
Maximum duration of sick leave within last 12 months	≥6 months	504	47.82
	3–6 months	126	11.95
	≤3 months	339	32.16
	None	65	6.17
	No employment	20	1.90
	Missing	0	0
Sick leave (admission)	Yes	617	58.54
	No	435	41.27
	Missing	2	0.19
Fit to work (discharge)	Yes	412	39.09
	No	635	60.25
	Not applicable	4	0.38
	Houseman/housewife	3	0.28
	Missing	0	0

### Clustering analyses

**FIGURE 2:**
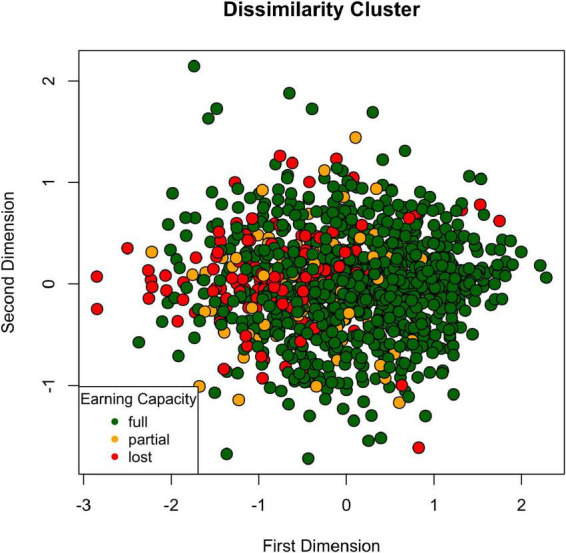
Dissimilarity cluster. Psychosomatic patients form a single cluster based on demographic, diagnostic, and psychological self-report data lining up on a polarized spectrum.

Non-parametric multidimensional scaling results based on Gower’s distance for mixed-type data are depicted in Figure 2. Patients form a single polarized cluster.

**FIGURE 3:**
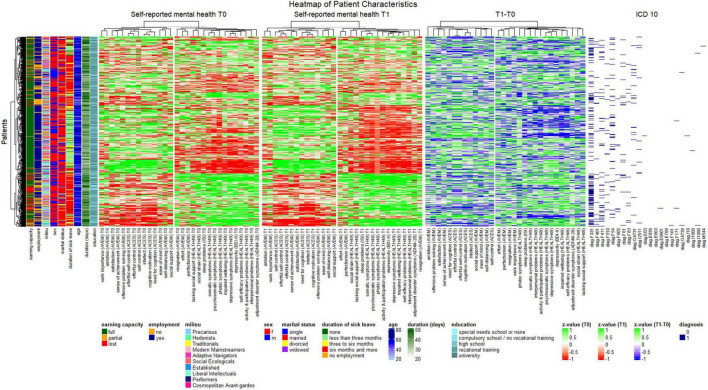
Heatmap of patient characteristics. Patients (rows) were clustered based on Gower’s distance, and numeric variables were k means clustered by time point.

Detailed clustering patterns are depicted in the heatmap (Figure 3). The optimal number of clusters as determined by silhouette method was *k* = 2 for all time points (Supplementary Figure 2). Measures of self-reported mental health were thus centered around two medoids. At both admission and discharge, one cluster encompassed psychosomatic pathology (HEALTH-49 scales, ADNM-20, BDI-II, ISI) alongside work-related measures resignation, perfectionism, and effort (AVEM), while the other cluster was comprised of cognitive effort investment scales (ACES) and the remaining measures of work-related behavior and experience (AVEM). Clustering of change patterns was identical except for AVEM scale work importance. As expected in a psychosomatic rehabilitation setting, the most common ICD-10 diagnoses in the data set were psychological and behavioral disorders such as depressive (F33.1 recurring depressive disorder) and anxiety disorders (F40.1 social phobias, F41.1 generalized anxiety disorder). Also, psychological and behavioral factors associated with disorders or diseases classified elsewhere (F54), non-organic sleep disorders (F51), obsessive-compulsive personality disorder (F60.5), mixed specific developmental disorders (F83) and mixed and other personality disorders (F61) were among the diagnoses with highest variance.

Patient dendrograms revealed three major groups, one of which was comprised almost exclusively of patients with full earning capacity, employment, a duration of sick leave of less than 3 months within the last year, comparatively low symptom load and positive work experience, and one with a majority of patients with a loss or reduction of earning capacity, unemployment, a duration of sick leave of more than 6 months, high symptom load and negative work experience. The latter was also characterized by recurrent depressive disorder, as well as, to a lesser degree, mixed specific developmental disorders and mixed and other personality disorders. A third group was best characterized by high unemployment and a duration of sickness absence of more than 6 months but included all assessments of earning capacity equally and revealed no clear pattern regarding symptom load and work experience and behavior.

### Classification analysis

**FIGURE 4:**
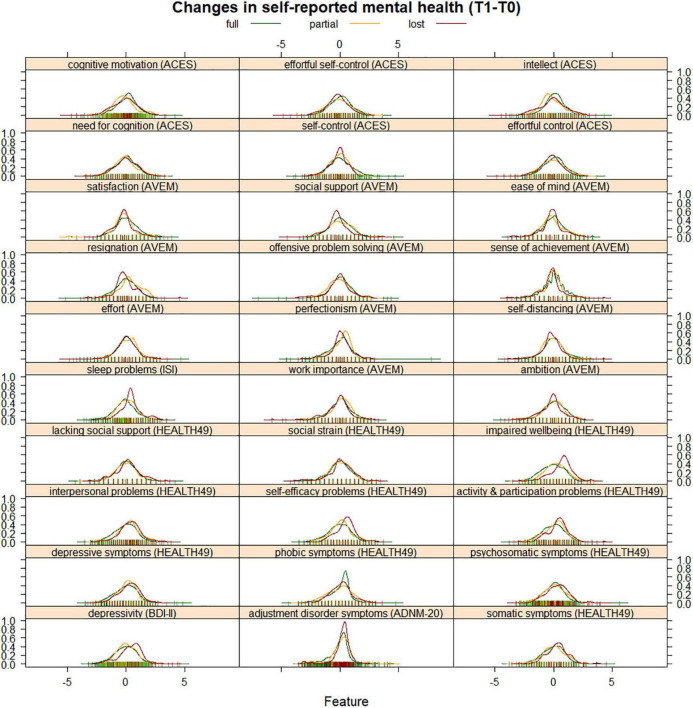
Density curves of change in psychological variables (T1-T0). Variables were scaled and centered for illustration. Values below zero indicate symptom improvement, and values above zero indicate symptom aggravation.

**FIGURE 5:**
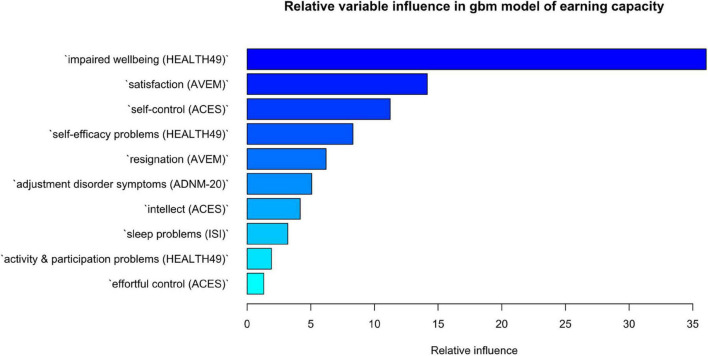
Relative influence of variables in gbm classification of earning capacity. The importance of a variable is the sum of empirical improvements by splitting that variable over each boosting iteration. The gbm algorithm implements Friedman’s extension to boosted models averaging the relative influence of a variable across all trees generated by the boosting algorithm.

The distribution of changes in self-reported mental health by earning capacity is shown in Figure 4. Values above zero indicate symptom aggravation, while values below zero show symptom improvement. Four variables had a mean correlation with other variables of *r* ≥ 0.7. These were depressivity (BDI-II), psychosomatic symptoms (HEALTH-49), and the cognitive motivation and effortful self-control scales (ACES), which were removed from the classification analysis. The best model tune for classifying patients by earning capacity was found for *n* = 50 trees, a minimum of *n* = 16 observations per node, an interaction depth of 1, and a shrinkage factor of 0.001, resulting in Kappa = 0.16 (*SD* = 0.0001). The confusion matrix of predicted and reference group membership is given in Table 2. The predicted model had a Kappa of 0.22. Variable influence in the model was identified to be highest for HEALTH-49 scale impaired wellbeing with a relative influence of 36.06 (Figure 5).

**TABLE 2:** Confusion matrix predicting earning capacity from changes in self-reported mental health.

	Reference		
	
Prediction	Full	Partial	Lost
full	252	1	88
partial	15	2	12
lost	19	0	32

### Regression analysis

Changes in impaired psychological wellbeing (HEALTH49) from T0 to T1 were chosen as outcome due to ranking highest in influence in the classification analysis of earning capacity. Interventions received during rehabilitation were entered as predictors. Optimal tuning parameters were found to be n = 250 trees, an interaction depth of 2, a shrinkage factor of 0.01 and a minimum of *n* = 2 observations per node. These resulted in a Root Mean Squared Error (RMSE) = 0.76 (*SD* = 0.03), *R*^2^ = 0.05 (*SD* = 0.007), and a Mean Absolute Error (MAE) = 0.60 (*SD* = 0.01). Running the model on the hold-out test set resulted in RMSE = 0.73, *R*^2^ = 0.04, and MAE = 0.59.

**FIGURE 6:**
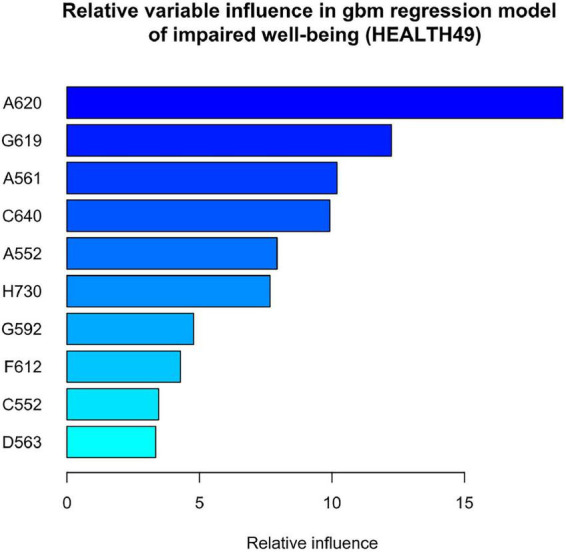
Relative influence of clinical interventions in gbm regression predicting change in impaired wellbeing (HEALTH49). A620 = physical exercise therapy with psychological goal setting, G619 = indicative behavioral group psychotherapy, A561 = weight machine muscle strength training in the group, C640 = seminar on disease-specific information, A552 = unmonitored group endurance training, H730 = ward group, G592 = individual behavioral psychotherapy, F612 = progressive muscle relaxation, C552 = medical consultation on disease and therapy, and D563 = social work consultation on professional perspectives.

**FIGURE 7:**
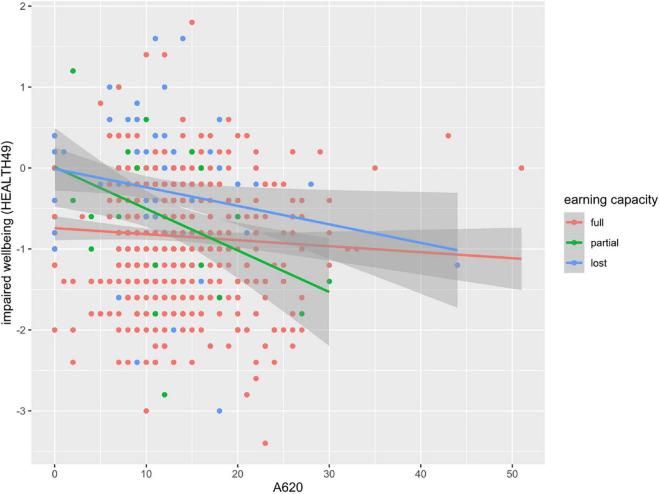
Regression plot of impaired wellbeing (HEALTH49) scores by units of intervention A620.

Variable influence was highest for A620 (physical exercise therapy with psychological goal setting), the top ten interventions with highest variable influence are shown in Figure 6, the regression plot for A620 is depicted in Figure 7.

## Discussion

We here presented a hypothesis-free analysis of clinical routine data aiming to elucidate the characteristics of patients with reduced earning capacity due to mental disorders. Demographic data revealed a gender bias with a higher proportion of female patients, which may reflect both females being disproportionately affected by internalizing disorders (30, 31), as well as men being less likely to seek treatment (32). Overall, we found a relatively heightened psychosomatic symptom load and reduced positive work experience, high unemployment, long sickness absence times and an accumulation of recurrent depressive disorder (F33.1), mixed specific developmental disorders (F83) and mixed and other personality disorders (F61) to be indicative of a potential reduction in earning capacity. Clustering patterns did not allow for distinction between partial and lost earning capacity.

Previous investigations support a link between impairments in earning capacity and duration of sickness absence (33), unemployment (34), and personality disorders (34, 35). The relationship between both sickness absence and unemployment and disability may seem self-evident at first, however, may reflect not only a logical contingency, but a self-reinforcing process. For instance, psychopathology may be aggravated by the lack of social contacts with colleagues, prospects of career and income progression, a sense of purpose, or daily routines and structures, thus eventually leading to chronification and disability.

We further found a subgroup of patients with mixed assessments of earning capacity, characterized by high unemployment and sickness absence times but no clear pattern of psychosomatic symptom load. These patients might be at heightened risk of deteriorating mental health in the future.

Improving the assessment of disability is a crucial goal considering the potential impact of alpha and beta errors. Adverse effects may range from symptom exacerbation through either work stress or failed adjustment to retirement, over an increase in treatment costs to unnecessary burden on a strained pension system. Indeed, receiving a disability pension itself may further lead to a deterioration in health due to altered alcohol and tobacco use, exercise, diet, or social isolation and even increase the risk of suicide (36, 37). Assessments of disability must therefore carefully balance the benefits and risks of supporting patients’ eligibility for disability pensions. We would thus like to stimulate more research into providing reliable and valid assessments of disability due to mental disorders. For instance, we suggest that the routine inclusion of evaluations according to the International Classification of Functioning, Health, and Disability (ICF), encompassing factors such as endurance, social skills, and organization skills among others, may dramatically improve the quality of disability assessments. Psychodiagnostic instruments are available in German (38) and English (39, 40).

Because assessment of earning capacity moreover depends on whether a patient could regain the ability to work, we focused our analysis on changes in psychological variables from admission to discharge. Interestingly, our data suggest that the quality of classification is moderate when it comes to determining future earning capacity from changes during rehabilitation. Direction of change may therefore be more informative than the extent of improvements during rehabilitation. While patients with reduced earning capacity showed a tendency for aggravation on most variables, the opposite was true for patients without disability. A notable exception were phobic symptoms. Considering that half of rehabilitants were on sick leave at the time of admission, heightened phobic symptoms in patients assessed to have full earning capacity may indicate anxieties associated with a prospective return to work (41). Meanwhile, changes in impaired psychological wellbeing had the highest relative influence on a split between all groups. The HEALTH-49 scale assesses positive feelings of relaxation, pleasure and comfort and is then recoded to indicate impairment. Its relative influence in the model may point to the importance of increasing positive experiences to preserve earning capacity in contrast to decreasing symptom load, a concept advocated by wellbeing therapy (42).

Lastly, we analyzed the influence of interventions on impaired wellbeing as the variable with the highest relative influence in distinguishing earning capacity. As expected, explaining 4–5% of the variance, the model accounted for a small portion of improvements in impaired wellbeing. This is not surprising given the time frame of 5 weeks and degree of chronification in the study group. On the one hand, the resulting model reflected the interplay of a multimodal treatment concept, on the other hand it identified exercise therapy with psychological goal setting to have the highest relative influence. Interestingly, regression lines showed a more pronounced decrease in impaired wellbeing with additional training units in patients with partial or lost earning capacity. This is surprising given the general tendency for aggravation in these groups. Further investigations might clarify whether an enhanced use of exercise therapy can help in the prevention of disability due to mental disorders. Research in other patient populations confirmed successful disease prevention. Exercise therapy has not only been found effective in improving wellbeing as assessed by the World Health Organization (43), but also in treating depression and preventing comorbid somatic illnesses, such as cardiovascular diseases, type 2 diabetes, and metabolic syndrome (44).

### Limitations

An important limitation of any machine learning analysis on naturalistic data is that results highly depend on the input. While diagnostic instruments used in this study are well-recognized and widespread among psychosomatic rehabilitation clinics, different sets of routine diagnostics may produce divergent results. Also, results reported here may reflect central and northern European countries but not generalize to other cultures and regions. In addition, treatment options in the clinical setting include both mandatory and facultative modules, which affect the variance in treatment units obtained and therefore the likelihood to vary with therapy outcomes. Intervention A620 included both mandatory and facultative treatment options, which may have both inflated the variance and caused a self-selection bias. Lastly, self-report measures may be subject to patient-specific effects, such as the tendency to exaggerate or understate symptoms depending on the desired assessment outcome.

## Data availability statement

The raw data supporting the conclusions of this article will be made available by the authors, without undue reservation.

## Ethics statement

Ethical review and approval was not required for the study on human participants in accordance with the local legislation and institutional requirements. Written informed consent for participation was not required for this study in accordance with the national legislation and the institutional requirements.

## Author contributions

LP and VK conceptualized the study. LP ran statistical analyses, generated figures, and wrote the manuscript. VK interpreted results and provided feedback on the manuscript. Both authors contributed to the article and approved the submitted version.

## Footnotes

1
https://bravors.brandenburg.de/de/verordnungen-215421

## Supplementary material

Supplementary Figure 1 Missingness structure. Missing data are colored in red, and complete data are colored in blue. The number of missing variable values is depicted in horizontal bar graphs, and the number of complete and incomplete patient responses is indicated in vertical bar graphs.

Supplementary Figure 2 Analysis of silhouette width to determine an optimal number of clusters.
