# Supplementary figures and images for: Using machine learning to investigate earning capacity in patients undergoing psychosomatic rehabilitation—A retrospective health data analysis
Source: Front Psychiatry. 2022 Oct 20;13:1039914. doi: 10.3389/fpsyt.2022.1039914 (PMC9630905; doi:10.3389/fpsyt.2022.1039914)

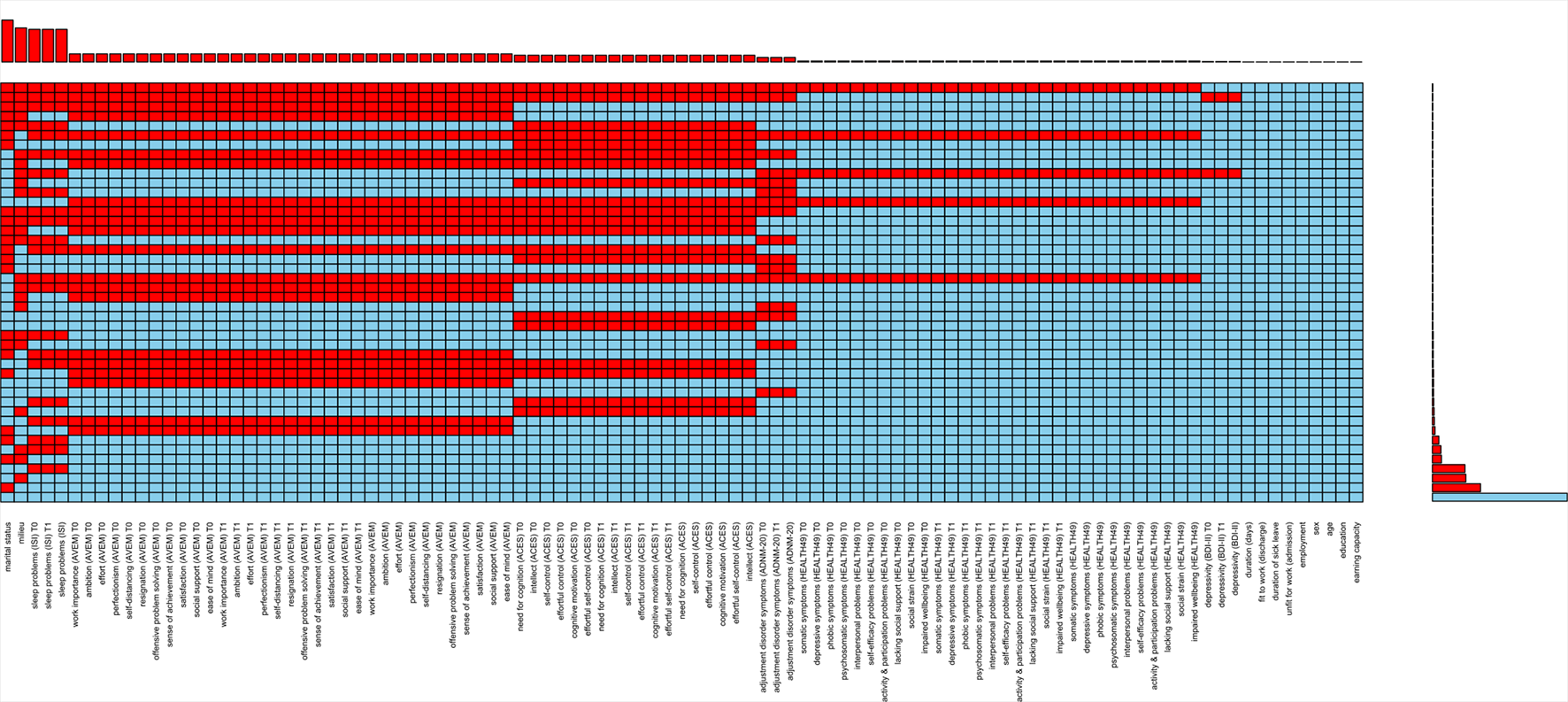

Supplement: Supplementary Figure 1 — Missingness structure. Missing data are colored in red, and complete data are colored in blue. The number of missing variable values is depicted in horizontal bar graphs, and the number of complete and incomplete patient responses is indicated in vertical bar graphs. [file Image_1.TIF]

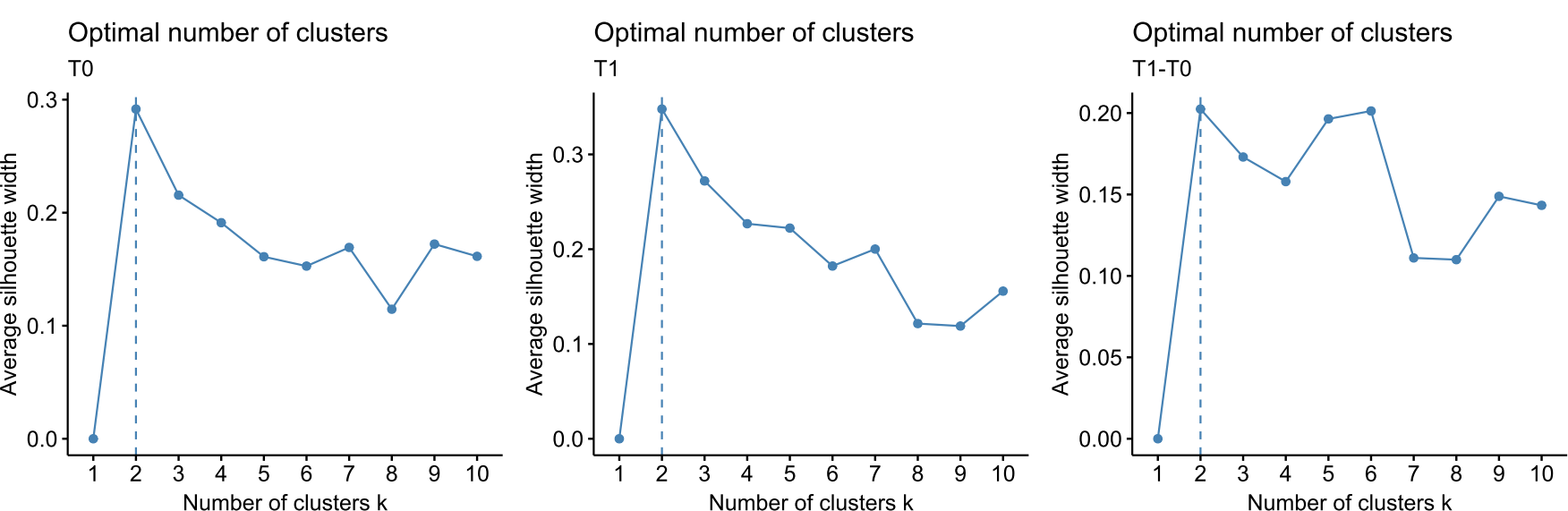

Supplement: Supplementary Figure 2 — Analysis of silhouette width to determine an optimal number of clusters. [file Image_2.TIF]
